# Redescriptions of *Nereis oligohalina* (Rioja, 1946) and *N. garwoodi* González-Escalante & Salazar-Vallejo, 2003 and description of *N. confusa* sp. n. (Annelida, Nereididae)

**DOI:** 10.3897/zookeys.518.9564

**Published:** 2015-08-25

**Authors:** Víctor M. Conde-Vela, Sergio I. Salazar-Vallejo

**Affiliations:** 1 El Colegio de la Frontera Sur, CONACYT, Departamento de Sistemática y Ecología Acuática, Chetumal, México

**Keywords:** Amphiamerican, taxonomy, estuarine nereidids, cryptic species, Polychaeta

## Abstract

Type material of several polychaete species described by Enrique Rioja from Mexican coasts are lost, and the current status of some species is doubtful. *Nereis oligohalina* (Rioja, 1946) was described from the Gulf of Mexico, but it has been considered a junior synonym of *Nereis occidentalis* Hartman, 1945, or regarded as a distinct species with an amphiamerican distribution. On the other hand, *Nereis garwoodi* González-Escalante & Salazar-Vallejo, 2003, described from Chetumal Bay, Caribbean coasts, could be confused with *Nereis oligohalina*. In order to clarify these uncertainties, *Nereis oligohalina* is redescribed based on specimens from the Mexican Gulf of Mexico, including a proposed neotype; further, *Nereis garwoodi* is redescribed including the selection of lectotype and paralectotypes, and *Nereis confusa* **sp. n.** is described with material from the Gulf of California. A key for the identification of similar species and some comments about speciation in nereidid polychaetes are also included.

## Introduction

Among the non-marine polychaetes, the family Nereididae de Blainville, 1818 has the largest number of brackish and freshwater species (61), and 31 out of these species occur in estuaries and coastal lagoons (Glasby et al. 2009). Of the 40 species of nereidid species recorded from the Gulf of Mexico (Fauchald and Solís-Weiss 2009), seven are reported in brackish or freshwater areas, and among the 10 species belonging to *Nereis*, only *Nereis oligohalina* (Rioja, 1946) is reported from estuaries (Glasby et al. 2009).

Enrique Rioja documented extensively the Mexican polychaetes from Pacific or Atlantic coasts in a series of papers; unfortunately, his material is lost, and most species require designation of neotypes (Salazar-Vallejo 1989). Rioja (1946) dealt with three estuarine nereidids from Veracruz, Mexico; he regarded one as a known species, *Neanthes succinea* (Leuckart, 1847), and the two others were described as new: *Neanthes oligohalina* and *Lycastopsis tecolutlensis*. The former species is now regarded as belonging in *Alitta*, but it differs from the North Sea species (T.F. Villalobos-Guerrero, pers. comm.); *Lycastopsis tecolutlensis* was regarded as a junior synonym of *Namanereis amboinensis* (Pflugfelder, 1933), nowadays a widespread species (Glasby 1999); and *Namanereis oligohalina* has been regarded as amphiamerican (Dean 2001), or restricted to Atlantic coasts (Santos and Lana 2003, Liñero-Arana and Díaz-Díaz 2007). However, other amphiamerican species have been shown to be restricted to one coast or the other, often resulting in description of new taxa (e.g. Carrera-Parra and Salazar-Vallejo 2011; Yáñez-Rivera and Carrera-Parra 2012). On the other hand, the Caribbean species *Namanereis garwoodi* González-Escalante & Salazar-Vallejo, 2003 could be confused with *Namanereis oligohalina*, and without an updated description of the latter, a synonymy can be anticipated.

However, *Nereis oligohalina* and *Nereis garwoodi* have morphological differences that separate them. In this contribution our objectives were first, to redescribe *Nereis oligohalina* based upon material collected from Veracruz, including topotypes, and to propose a neotype. Second, to redescribe *Nereis garwoodi* to clarify some doubtful features in the original description, and to select lectotype and paralectotypes specimens from the syntype series. Third, to recognize what has been regarded as *Nereis oligohalina* from the Mexican Pacific as a distinct species and describe it as *Nereis confusa* sp. n., based upon material from the Gulf of California. Further, a key to identify similar *Nereis* species and comments about species delimitation are also included.

## Material and methods

Specimens studied are deposited in the Reference Collection of El Colegio de la Frontera Sur, Chetumal (ECOSUR) including ethanol-fixed specimens (ECOSUR-OH), and in the Polychaetological Collection of the Universidad Autónoma de Nuevo León (UANL).

Topotypes of *Nereis oligohalina* from Estero Casitas, Nautla were examined, including additional specimens that were recently collected along the coast of Veracruz, Mexico, Gulf of Mexico. Specimens of *Nereis confusa* sp. n. from Bahía La Paz and Bahía de Los Ángeles, Gulf of California were found in unidentified material in ECOSUR, now formally deposited. To assess variation in paragnath morphology, specimens of *Nereis pelagica* Linnaeus from England (ECOSUR P2840), and *Pseudonereis* sp. from the Caribbean Sea (ECOSUR P1170), were also examined.

The best preserved specimens were used for designation of type material. Some specimens were fixed and preserved in 96% ethanol directly, otherwise the specimens were fixed with formalin and later preserved in 70% ethanol.

For analysis of variation, type materials and a number of non-type specimens for each species were measured. Total body length (TL), length up to chaetiger 3 (L_3_) or 10 (L_10_), width at same chaetigers (W_3_ and W_10_), number of chaetigers (nC), and length of longest tentacular cirrus (rTC) were measured with a millimeter rule under the stereomicroscope. TL was measured from palp tips to the end of the pygidium, W_3_ and W_10 _were measured excluding parapodia. Also, paragnath numbers in all areas were counted; if the pharynx was not everted, a ventral dissection was made, and areas VII-VIII were described as if the pharynx was exposed. With these results simple descriptive statistics (mean, range and standard deviation) were performed. As different fixation methods were used, a Mann-Whitney U-test was used for evaluating if there were significant differences in body measures as a result of fixation method.

For the microscopical observation of parapodial features and chaetae, right-side parapodia along body were removed and mounted in semi-permanent slides; the photographs were made with a digital camera, and distal-view drawings of parapodia were included to depict spatial disposition of ligules and chaetae. Descriptions of pigmentation patterns were included, because they are consistent and useful for recognizing the three species; other authors have noted their utility for identifying cryptic nereidid species (Read 2007, Glasby et al. 2013).

Bakken and Wilson’s (2005) terminology was followed for describing parapodia, and Bakken et al. (2009) for paragnaths. Parapodia from both atokes and epitokes (if available) were illustrated to show parapodial changes along the body. For determining dorsal cirri length and position of attachment, we considered the beginning of the dorsal or notopodial ligule to be approximately at the same vertical position of attachment as the ventral cirri; therefore, the relative length of dorsal cirrus was measured from that position toward the distal end of dorsal or notopodial ligule. The dorsal cirrus was considered basally attached if placed at, or near such a position, or medially attached if it was displaced more distally from that position.

Also, the reach of the dorsal cirrus and its relative length in respect of the dorsal or notopodial ligules were considered as separate attributes. For determining the reach of the dorsal cirrus, the tips of the both dorsal cirrus and dorsal or notopodial ligules were taken into account; if the dorsal cirrus tip extended beyond the tip of the dorsal or notopodial ligule, then we report “dorsal cirrus extended beyond dorsal/notopodial ligule” rather than “dorsal cirrus longer than dorsal/notopodial ligule”. On the contrary, if dorsal cirrus is shorter or not exceeding the dorsal or notopodial ligules, then we report “dorsal cirrus not extended beyond dorsal/notopodial ligule”. In the species herein treated, length of dorsal cirri and length of dorsal or notopodial ligules were generally subequal, and the dorsal cirri change their attachment along the body, but not their length necessarily.

For the designation of lectotype and neotype, the International Code of Zoological Nomenclature (ICZN 1999) was followed. The designation of a neotype for *Nereis oligohalina* follows Article 75, and the designation of a lectotype for *Nereis garwoodi* follows Article 74 (ICZN 1999). The non-formal term ‘paraneotypes’ is used for figured topotypic specimens (Evenhuis 2008), and their utility has been pointed out elsewhere (Salazar-Vallejo 2011, Sendall and Salazar-Vallejo 2013).

## Results

### Family Nereididae de Blainville, 1818

#### Genus Nereis

Taxon classificationAnimaliaPhyllodocidaNereididae

Linnaeus, 1758

##### Type species.

*Nereis pelagica* Linnaeus, 1758, by subsequent designation (Hartman 1948:63).

##### Remarks.

Linnaeus (1758:654) listed five species under *Nereis*: *Nereis lacustris* (now *Stylaria lacustris*, an oligochaete), *Nereis caerulea* (questionable after Hartman 1959:254), *Nereis gigantea* (after Hartman 1959:259, same as *Hermodice carunculata* (Pallas, 1766), rendering it a *nomen oblitum* because it would have priority over Pallas’ name), *Nereis pelagica*, and *Nereis noctiluca*. Further, it was Hartman (1948:63) who fixed the type species, and therefore this should be regarded as a subsequent designation (ICZN 1999, Art. 69.1), in contrast to Bakken and Wilson (2005) who regarded it as an original designation.

Although the species described here have more attributes than those included in the current diagnosis of the genus (Bakken and Wilson 2005), the generic diagnosis was not modified because it first requires a redescription of the type-species, and a phylogenetic analysis with subsequent delimitation of the genus. Among the traditionally used features for descriptions and delimitation of *Nereis* species are some that are highly variable, especially paragnath number. Bakken et al. (2009) made a useful revision of paragnath morphology and introduced new terminology to standardize descriptions. In addition to conical paragnaths, the species described here present other types of paragnaths that are not currently included in the diagnosis of the genus. Conical paragnaths are pointed to various degrees, being more acute in the maxillary ring, especially on area II. The pyramidal paragnaths in *Nereis oligohalina* and *Nereis garwoodi* have quadrilateral bases but they can also be polygonal, having more defined surfaces in the latter species. Further, *Nereis confusa* sp. n. apparently has smooth bars on area IV, but a closer inspection confirms that this is an artifact because the bars are formed by lateral and basal fusions of some small conical paragnaths (Fig. 6H); these modified structures were regarded as melted paragnaths (Bakken et al. 2009). However, Glasby et al. (2011) suggested limiting use of the term for conical paragnaths mounted on a plate-like basement as occurs in *Neanthes pachychaeta* (Fauvel, 1918), and Villalobos-Guerrero and Carrera-Parra (2015) found paragnaths on a soft basement in *Alitta acutifolia* (Ehlers, 1901). Because neither a basement is present in *Nereis confusa* sp. n., we suggest the term ‘merged’ for paragnaths fused at the base but without formation of a plate.

In his revision of *Pseudonereis* Kinberg, 1865, Bakken (2007) introduced the term ‘P-bar’, which was later defined by Bakken et al. (2009) as “small bars having a protruding apex in one end of the bar”; and they can appear in areas II, III, IV and VII-VIII, often accompanied by conical paragnaths. The monophyly of *Pseudonereis* was supported by, among other characters, the presence of both P-bars and paragnaths in comb-like rows (Bakken 2007). The *Nereis* species studied herein have two main rows, each one with other two sub-rows; the anterior-most sub-rows are often aligned horizontally, while the posterior-most ones form a jagged line. The anterior-most rows have P-bars alternating with conical or pyramidal paragnaths in a similar way as in *Pseudonereis* (Fig. 6D–F), which has been also reported for *Alitta* (Villalobos-Guerrero and Carrera-Parra 2015). Therefore, P-bars are not an exclusive feature of *Pseudonereis* as Bakken et al. (2009) concluded.

The neuropodial postchaetal lobe has been considered absent for *Nereis* species in recent phylogenetic analyses (Bakken and Wilson 2005, Santos et al. 2005). Nevertheless some authors indicate its presence; Read (1980) for *Nereis ovarius* (Read), Santos and Lana (2003) for *Nereis pseudomoniliformis* (Santos and Lana), Chambers and Garwood (1992) for *Nereis pelagica* (and corroborated by us), and Darbyshire (2014) for *Nereis eugeniae* (Kinberg, 1865). Also, the three species herein described have postchaetal lobes shorter or subequal than neuroacicular ligules, and in epitoke specimens these lobes carry natatory lamellae; therefore, we considered postchaetal lobes as present in *Nereis*.

Reproductive nereidids or epitokes can have two or three different regions; parapodial cirri and the pygidium are transformed is especially relevant for chemorreception, parapodial lobes or ligules are expanded and chaetae replaced for swimming (Herpin 1925, Boilly-Marer 1972). Charrier (1920) studied muscular tissue transformation associated with epitoky in the commensal species *Nereis fucata* (Savigny *in* Lamarck, 1818) (currently belonging to *Neanthes* Kinberg). He observed that parapodial cirri vary in some features as the length relative to corresponding ligules, the modifications of attachment and the displacement along the body; in fact, the attachment site of dorsal cirrus often show a distal displacement along notopodial ligules.

#### Nereis oligohalina

Taxon classificationAnimaliaPhyllodocidaNereididae

(Rioja, 1946)

**Figure 1.**
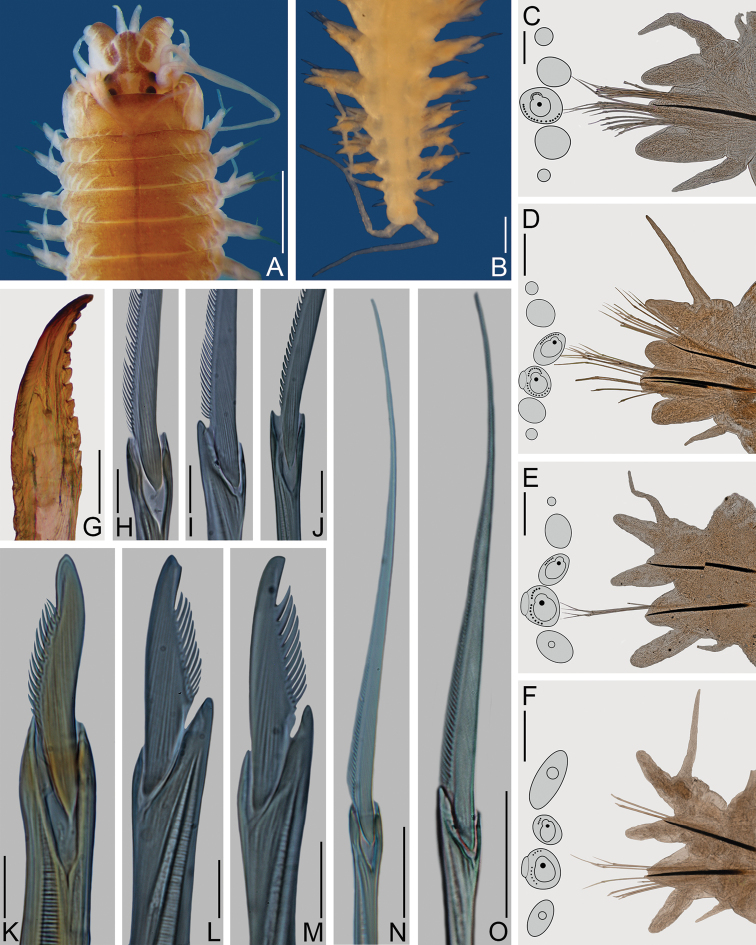
*Nereis oligohalina*. Neotype female **A–G** (ECOSUR 0172); paraneotypes **H–O** (ECOSUR 0173). **A** Anterior end, dorsal view **B** Posterior end, dorsal view **C** Parapodium 2, anterior view **D** Parapodium 10, anterior view **E** Parapodium 46, anterior view **F** Parapodium 64, anterior view **G** Left jaw, dorsal view **H** Supra-acicular homogomph spiniger, parapodium 40 **I** Sub-acicular heterogomph spiniger, from same **J** Supra-acicular homogomph spiniger, from same **K** Notopodial homogomph falciger, from same **L** Supra-acicular heterogomph falciger, parapodium 28 **M** Sub-acicular heterogomph falciger, from same **N** Notopodial homogomph spiniger, parapodium 40 **O** Sub-acicular heterogomph spiniger, from same. Scale bars: 1 mm (**A**); 0.3 mm (**B, G**); 50 μm (**C**); 0.1 mm (**D–F**); 10 μm (**H–M**); 30 μm (**N, O**).

**Figure 2.**
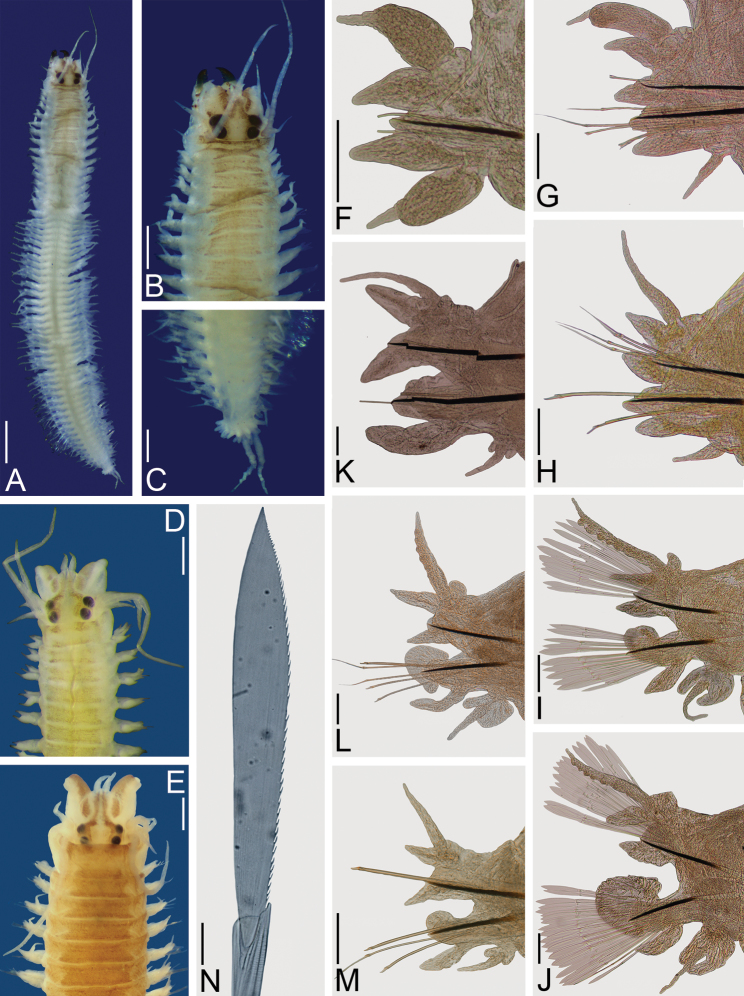
*Nereis oligohalina*. Non-type male **A–C, F–J, N** (ECOSUR–OH–P0761); non-type partially transformed male **D, L–M** (ECOSUR P2827); non-type partially transformed female **E, K** (ECOSUR P2827). **A** Whole specimen, dorsal view **B** Anterior end, dorsal view **C** Posterior end, dorsal view **D, E** Anterior ends, dorsal view **F** Parapodium 1, anterior view **G** Parapodium 6, anterior view **H** Parapodium 10, frontal view **I** Parapodium 18, anterior view **J** Parapodium 31, anterior view **K** Parapodium 10, anterior view **L** Parapodium 24, anterior view **M** Parapodium 52, anterior view **N** Sesquigomph natatory chaetae, parapodium 31. Scale bars: 1 mm (**A, D–E**); 0.5 mm (**B**); 0.2 mm (**C**); 0.1 mm (**F–M**); 10 μm (**N**).

Figures 1 , 2 , 6A, D, J–L, O, Q

Neanthes oligohalina Rioja 1946: 207–210, pl. 1, figs 3–6, pl. 2, figs 13–19; 1947: 529, 531; 1960: 295.
Nereis oligohalina Hartman 1951: 46; 1954: 414.

##### Type material.

**Veracruz, Mexico.** Neotype ECOSUR 0172 and paraneotypes ECOSUR 0173 (5), mouth of Actopan River (19°25'2.95"N, 96°19'32.28"W), Chachalacas Sandbar, Gulf of Mexico, 1 m depth, on *Crassostrea virginica* reef, fine sediment, April 10 2012, Coll. V.M. Conde-Vela, A.E. Te-Gómez.

##### Additional material.

**Veracruz, Mexico.** ECOSUR P2826 (15), and ECOSUR P2827 (37), Mouth of Actopan River (19°25'2.95"N, 96°19'32.28"W), Chachalacas Sandbar, Gulf of Mexico, 1 m depth, on *Crassostrea virginica* reef, fine sediment, April 10 2012, Coll. C. Licona-Rosado, V.M. Conde-Vela, A.E. Te-Gómez. ECOSUR P2828 (1) Laguna de Alvarado, St. 8 (18°45'20.34"N, 95°46'29.04"W), December 6 2012, in rocks, Coll. J.M. Aguilar-Camacho. ECOSUR-OH-P0760 (23), Las Barrillas, St. 4 (18°11'20.15"N, 94°35'56.97"W), December 5 2012, on *Crassostrea virginica* culture, Coll. J. Cruz-Terrón. ECOSUR-OH-P0761 (15), Laguna Grande, Mandinga, St. 28 (19°1'54.96"N, 96°4'8.10"W), December 10 2012, on oyster, 11.41‰, 27.24 °C, Coll. T.F. Villalobos-Guerrero, MA. Tovar-Hernández, J.M. Aguilar-Camacho. ECOSUR-OH-P0762 (3), Laguna Grande, Mandinga, St. 26 (19°2'20.64’’ N 96°4'24.24"W), December 10 2012, on mangrove and oyster, 10.74 ‰, 26.63 °C, Coll. J.M. Aguilar-Camacho, T.F.Villalobos-Guerrero. UANL-3918 (6), Estero Casitas, Nautla, March 25 1990, Coll. A. Contreras-Arquieta.

##### Neotype locality.

Mouth of Actopan River (19°25'2.95"N, 96°19'32.28"W), Gulf of Mexico, in *Crassostrea virginica* (Gmelin) reef, in muddy sediment, 1 m depth.

##### Description.

Neotype complete (ECOSUR 0172), atokous female. Body tapering, 38 mm long, 2.1 mm wide, 74 chaetigers, filled with oocytes. Body yellowish, reddish brown pigmentation present dorsally on first quarter of body, discoloring towards midbody chaetigers; lateral oblique pale lines along chaetigers 1–9 (Fig. 1A), replaced by fingerprint-like lines from chaetiger 10 (Fig. 6L), anterior margin of segments with thin transverse band (Fig. 1A). Prostomium with pigmentation reddish brown along inner half of palps and around eyes; two broad hourglass-shaped lines extending from antennae towards eyes, separated by a thin pale line and two oval patches, one on each side of darker areas (Fig. 1A). Peristomium pigmented, pale lines present (Fig. 1A).

Prostomium 1.5 times longer than wide; antennae cirriform, slightly passing palps; eyes subequal, black, in trapezoidal arrangement (Fig. 1A). Peristomium three times longer than first chaetiger; tentacular cirri with short ceratophores; dorsal cirri longer than ventral ones, longest posterodorsal cirri reaching chaetiger 6 (Fig. 1A).

Pharynx dissected; jaws light brown with 11 rounded teeth, extending to base (Fig. 1G). Maxillary ring: I = 12 cones in triangle, II = 32–31 cones in arc, III = 50 cones in an ellipse, IV = 34–32 cones in arc. Oral ring: V = 1 cone, VI = 4–4 pyramids in diamond, VII-VIII = 42 in two irregular rows, P-bars alternating with small pyramids in anterior-most row, similar-sized pyramids alternating in posterior-most row (in everted pharynx).

Parapodial cirri pattern: Dorsal cirri longer than upper dorsal ligules throughout body; basally inserted on anterior region, displaced medially on midbody and posterior chaetigers. Ventral cirri shorter than neuropodial ligules throughout body, longer in few anterior chaetigers, basally inserted on anterior region, progressively distant throughout body.

First two chaetigers uniramous, remaining ones biramous. Uniramous parapodia (Fig. 1C) with dorsal cirri basal, slightly longer than dorsal ligules. Dorsal ligules digitate; neuroacicular ligules subconical, subequal to dorsal ones; neuropodial ventral ligules digitate, slightly longer and basally twice as broad as dorsal ones. Ventral cirri slightly shorter than neuropodial ventral ligules; both dorsal and ventral cirri with similar width.

In anterior parapodia (Fig. 1D), dorsal cirri medial, slightly longer than notopodial dorsal ligules, extending beyond their tips. Notopodial dorsal ligules subconical; subequal to ventral ones; notopodial ventral ligules globose, notoacicular papillae conspicuous. Neuroacicular ligules globose, postchaetal lobes rounded, slightly shorter than neuroacicular ligules; neuropodial ventral ligules digitate, slightly shorter than neuroacicular ones. Ventral cirri shorter than neuropodial ligules; both dorsal and ventral cirri with similar width.

In middle and posterior parapodia (Fig. 1E, F), dorsal cirri medial, slightly shorter than notopodial dorsal ligules. Notopodial dorsal and ventral ligules subequal, subconical, longer than wide, notoacicular papillae conspicuous in middle parapodia only. Neuroacicular ligules subconical, wider than long, postchaetal lobes rounded, about half as long as neuroacicular ligules; neuropodial ventral ligules digitate, 3–4 times longer than wide, medially attached to neuroacicular ligules, 2–3 times longer than them. Ventral cirri half as long or one-third as long as neuropodial ventral ligules; dorsal and ventral cirri with similar width. Glandular masses slightly conspicuous on ligules in posterior parapodia (Figs 1F, 6Q).

In anterior and midbody parapodia notochaetae homogomph spinigers; neurochaetae homogomph spinigers and heterogomph falcigers in supra-acicular fascicles, heterogomph spinigers and falcigers in sub-acicular fascicles. In posterior parapodia, notochaetae homogomph spinigers and falcigers; neurochaetae as in anterior parapodia. Chaetae decreasing rapidly in number toward posterior end.

Notopodial homogomph spinigers pectinate (i.e. blade narrow with parallel teeth), teeth decreasing distally (Fig. 1N). Notopodial homogomph falcigers with sigmoidal blade, pectinate, distal tooth recurved, fused to blade (Fig. 1K). Neuropodial homogomph spinigers pectinate (Fig. 1H) or basally serrated (i.e. blade small with coarse teeth) (Fig. 1J), heterogomph spinigers pectinate (Fig. 1I); spinigers of similar size, teeth decreasing in size distally (Fig. 1N, O). Neuropodial heterogomph falcigers pectinate, distal tooth recurved, fused to blade, supra-acicular falcigers slightly broader than sub-acicular ones (Fig. 1L, M).

Pygidium not modified; anal cirri cirriform, as long as last 5–6 chaetigers (Fig. 1B).

##### Epitokes.

Male fully transformed (ECOSUR-OH-P0761) complete; body tapering, 9 mm long, 0.9 mm wide, 57 chaetigers. Partially transformed male (ECOSUR P2827) complete; body tapering, 38 mm long, 2.1 mm wide, 57 chaetigers. Partially transformed female (ECOSUR P2827) complete; body tapering, 20 mm long, 1.6 mm width, 55 chaetigers. All with body yellowish with brown pigmentation present dorsally on first quarter of body, discoloring towards midbody chaetigers; faint lateral lines (Fig. 2A, D, E). Prostomium with pigmentation as in atokes, but less intense (Fig. 2B).

Prostomium longer than wide; antennae cirriform, slightly wider than those present in atokous female, as long as palps; eyes subequal, two (Fig. 2D) or three times (Fig. 2B) larger than antennal basal width, black, in trapezoidal arrangement (Fig. 2B, D).

Peristomium twice as long as first chaetiger, slightly pigmented; tentacular cirri present; dorsal tentacular cirri longer than ventral ones, posterodorsal ones reaching to chaetiger 10 (Fig. 2B, D).

Fully transformed male with pharynx everted, jaws amber with 10 teeth, inner edge toothed throughout. Maxillary ring: I = 8 cones in triangle, II = 30–32 cones in arc, III: 40 cones in rectangle, IV: 28–28 pointed cones in arc. Oral ring: V = 1 cone, VI: 4–4 pyramids in diamond, VII-VIII: 46 in two irregular rows, pyramids alternating with small cones in most-anterior row, pyramids with similar size alternating in most-posterior row.

Male body divided into two regions (Fig. 2A). Pre-natatory region includes chaetigers 1–16, natatory region from chaetiger 17 to end of body. Partially transformed female divided into two inconspicuous regions, parapodial lamellae visible from chaetiger 24.

Parapodial cirri pattern: Anterior parapodia with dorsal cirri modified in chaetigers 1–7 in males, 1–5 in females; ventral cirri modified in chaetigers 1–5 in males, 1–4 in females; modification attenuated in females. Dorsal cirri subequal to upper dorsal ligules in anterior chaetigers, slightly longer throughout body; basally inserted in anterior-most region, displaced medially toward end of body. Ventral cirri shorter than neuropodial ventral ligules in unmodified chaetigers, subequal in modified region; basally inserted in anterior region, barely displacing ventrally throughout body.

Chaetigers 1–2 uniramous (Fig. 2F); modified dorsal cirri basal, subpyriform (i.e. basally broad, medially broader, distally narrow), slightly longer than dorsal ligules. Dorsal and neuropodial ventral ligule subequal, subconical. Neuroacicular ligule subconical, much shorter than ventral one; postchaetal lobes rounded. Modified ventral cirri subpyriform, subequal to neuropodial ventral ligule; both dorsal and ventral cirri with similar width.

**Figure 3.**
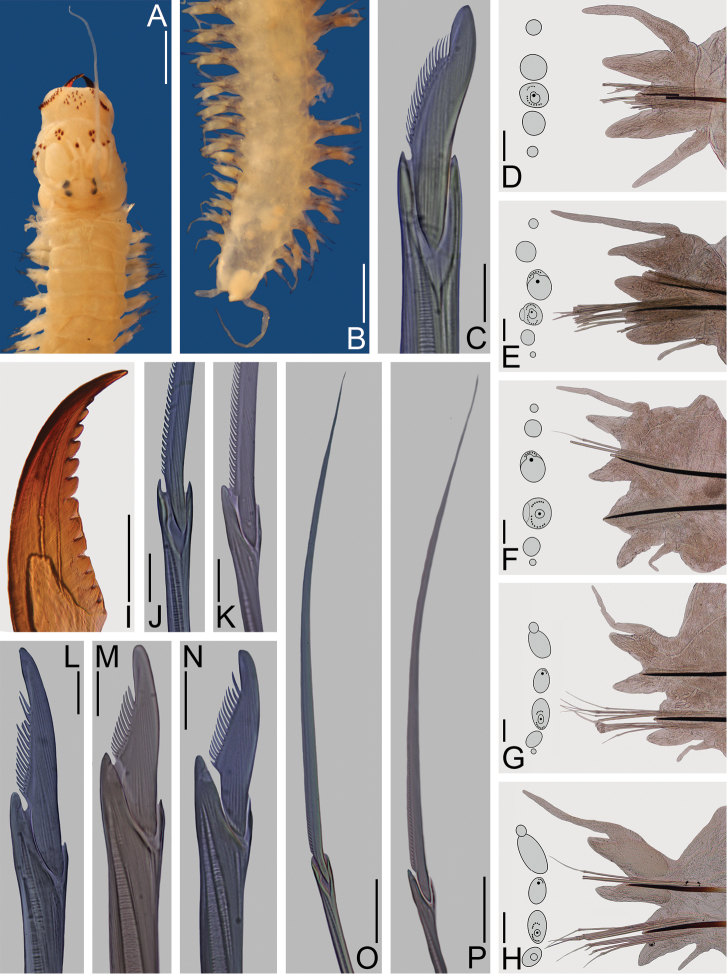
*Nereis garwoodi*. Lectotype female **A–B, D–P** (ECOSUR 0065); paralectotype **C** (ECOSUR 0066). **A** Anterior end, dorsal view **B** Posterior end, dorsal view **C** Notopodial homogomph falciger, parapodium 75 **D** Parapodium 2, anterior view **E** Parapodium 9, anterior view **F** Parapodium 28 **G** Parapodium 56, anterior view **H** Parapodium 80, anterior view **I** Left jaw, dorsal view **J** Supra-acicular homogomph spiniger, parapodium 80 **K** Sub-acicular heterogomph spiniger, from same **L** Supra-acicular heterogomph falciger, parapodium 56 **M** Supra-acicular heterogomph falciger, parapodium 80 **N** Sub-acicular heterogomph falciger, from same **O** Supra-acicular heterogomph spiniger, from same **P** Sub-acicular heterogomph spiniger, from same. Scale bars: 1 mm (**A–B, I**); 50 μm (**C, J–N**); 0.1 mm (**D–H**); 0.3 mm (**O–P**).

**Figure 4.**
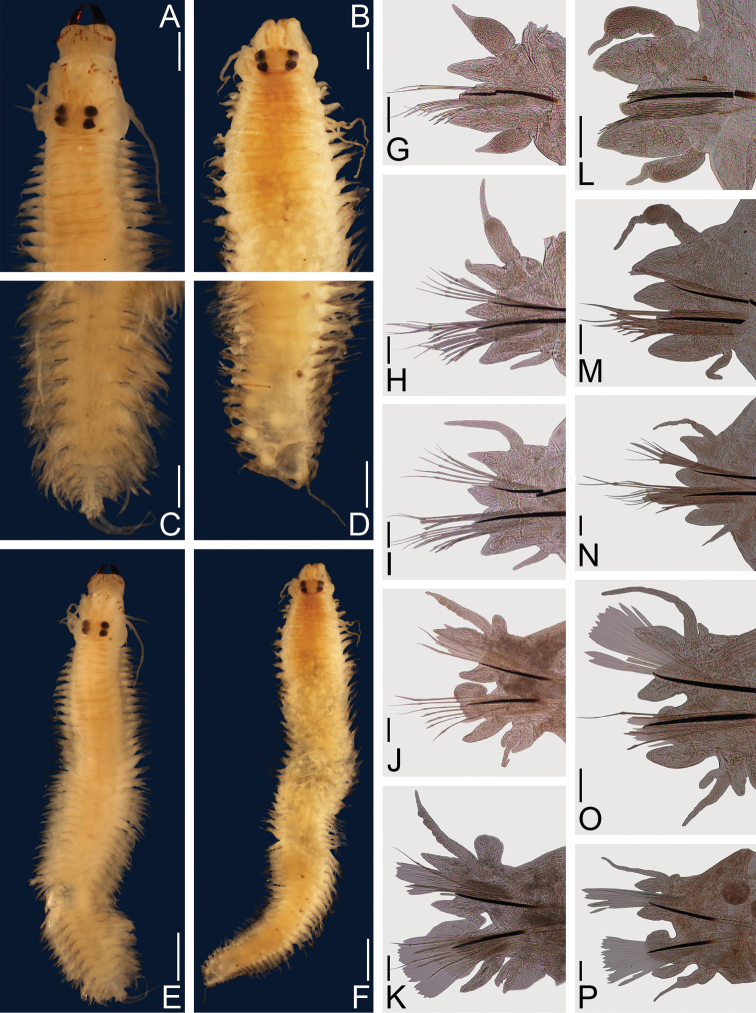
*Nereis garwoodi*. Paralectotype male **A, C, E, G–K** (ECOSUR 0066); paralectotype female **B, D, F, L–P** (ECOSUR 0066). **A, B** Anterior ends, dorsal view. **C, D** Posterior ends, dorsal view **E, F** Whole specimens, dorsal view **G** Parapodium 2, anterior view **H** Parapodium 6, anterior view **I** Parapodium 10, anterior view **J** Parapodium 18, anterior view **K** Parapodium 36, anterior view **L** Parapodium 2, anterior view **M** Parapodium 5, anterior view **N** Parapodium 10, anterior view **O** Parapodium 26, anterior view **P** Parapodium 36, anterior view. Scale bars: **A–D** = 0.5 mm; **E–F** = 1 mm; **G–P** = 0.1 mm.

**Figure 5.**
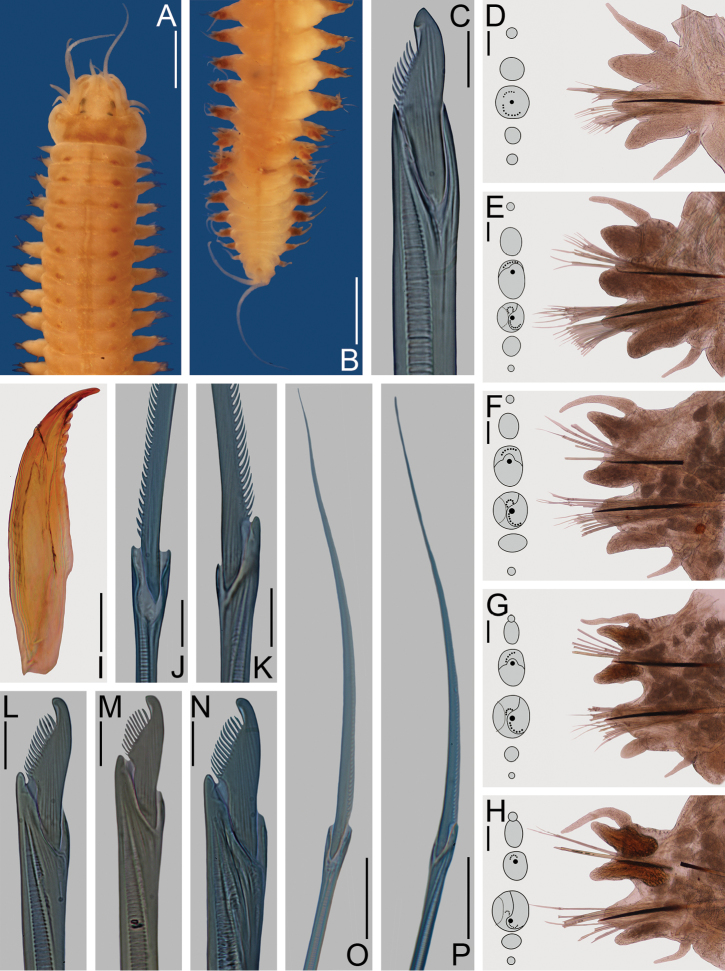
*Nereis confusa* sp. n. Holotype **A–P** (ECOSUR 0174). **A** Anterior end, dorsal view **B** Posterior end, dorsal view **C** Notopodial homogomph falciger, parapodium 72 **D** Parapodium 2, anterior view **E** Parapodium 11, anterior view **F** Parapodium 28, anterior view **G** Parapodium 51, anterior view **H** Parapodium 72, anterior view **I** Left jaw, dorsal view **J** Supra-acicular homogomph spiniger, parapodium 51 **K** Sub-acicular heterogomph spiniger from same **L** Sub-acicular heterogomph falciger from same **M** Supra-acicular heterogomph falciger, parapodium 72 **N** Supra-acicular heterogomph falciger from same **O** Supra-acicular homogomph spiniger from parapodium 51 **P** Sub-acicular heterogomph spiniger from same. Scale bars: 1 mm (**A–B**, **I**); 50 μm (**C, J–N**); 0.1 mm (**D–H**); 0.3 mm (**O–P**).

Chaetigers 3–7 in males (Fig. 2G) and 3–5 in females, with slightly modified biramous parapodia. Modified dorsal cirri medial, cattail-like (i.e. basal section broad, long; cirrostyle markedly narrower), slightly longer than notopodial dorsal ligules, extending beyond them; basal section 2–3 longer than distal one. Notopodial dorsal ligules subconical, subequal to notopodial ventral one; notopodial ventral ligule subconical, notoacicular papilla conspicuous. Neuroacicular ligule subconical, postchaetal lobe rounded, shorter than neuroacicular ligule; neuropodial ventral ligule digitate, as long as neuroacicular one (Fig. 2G). Modified ventral cirri cattail-like, subulate in chaetigers 6–7 in males and 5 in females, shorter than neuropodial ventral ligules; modified dorsal and ventral cirrus with similar width.

Chaetigers 8–16 in males and 6–23 in females with parapodial proportions as in atokes, but with more acute ligules (Fig. 2H).

Remaining parapodia biramous, modified (Fig. 2I, J, L, M). Dorsal cirri medial, subulate, ventral margins sinuate in males only, longer than notopodial ligules; basal lamellae small, smallest in females, progressively smaller toward posterior region. Notopodial dorsal and ventral ligules subequal, subconical, notoacicular papillae conspicuous; ventral ligules with lamellae without projections along ventral margins. Neuroacicular ligules subconical, subequal to notopodial ventral ones; postchaetal lobes developing into flabellate lamellae, increasing in size posteriorly, decreasing in posterior-most chaetigers, some with slight projections in dorsal or ventral edge; neuropodial ventral ligules digitate, medially attached to neuroacicular ones. Ventral cirri subulate, subequal to neuroacicular ligules, with two basal lamellae of different sizes; dorsal cirri wider than ventral ones.

Prenatatory region with noto- and neurochaeta as in atokes, homogomph falcigers not observed, blades missing in most chaetae, number progressively reduced. In natatory region, noto- and neurochaetae sesquigomph chaetae with finely serrated, paddle-like blades (Fig. 2N).

In fully transformed males pygidium with anus surrounded by rosette of papillae (Fig. 2C); anal cirri sinuous, as long as last 5–6 chaetigers (Fig. 2C).

Transformation in females discrete; small lamellae on base of dorsal cirri, neuroacicular ligules and ventral cirri (Fig. 2K); size of lamellae increasing toward posterior end and becoming inconspicuous in far posterior chaetigers; other features as in atokous female.

##### Variation.

The results of the analysis of body variation and paragnath numbers are summarized in Tables 1 and 2. The effect of fixation techniques on the shape of specimens have been recently evaluated by Oliveira et al. (2010) for *Laeonereis acuta* (Treadwell, 1923). The authors concluded that techniques of fixation can influence the shape and body proportions, especially if specimens were not previously relaxed, leading to erroneous identifications.

**Table 1.** Ranges, means and standard deviation of some body measures in three *Nereis* species (TL: total length, L3 and L10: length at chaetiger 3 and 10, respectively; W3 and W10: width at chaetiger 3 and 10, respectively; nC: number of chaetigers; rTC: reach of largest tentacular cirrus; SD: standard deviation).

*Nereis oligohalina*		TL	L_3_	L_10_	W_3_	W_10_	nC	rTC
Formalin specimens (n = 23)	Minimum	10.0	1.1	1.6	0.6	0.6	55.0	4
	Maximum	40.0	3.0	6.3	2.2	1.9	80.0	7
	Mean	24.8	2.3	3.8	1.6	1.4	66.9	5.7
	SD	9.3	0.5	1.3	0.5	0.4	7.8	0.9
Ethanol specimens (n = 19)	Minimum	9.0	0.6	1.5	0.7	0.6	57.0	5
	Maximum	36.0	3.0	6.5	2.2	2.0	78.0	14
	Mean	20.4	1.5	3.5	1.4	1.3	67.0	9.3
	SD	9.3	0.6	1.3	0.5	0.5	8.8	2.5
***Nereis garwoodi***		**TL**	**L_3_**	**L_10_**	**W_3_**	**W_10_**	**nC**	**rTC**
Formalin specimens (n=33)	Minimum	9.0	0.7	2.4	1.0	0.8	46.0	6
	Maximum	36.0	3.0	6.9	2.0	2.1	96.0	13
	Mean	22.1	1.8	3.8	1.5	1.5	72.7	9.3
	SD	9.4	0.5	1.0	0.3	0.3	11.8	1.5
***Nereis confusa* sp. n.**		**TL**	**L_3_**	**L_10_**	**W_3_**	**W_10_**	**nC**	**rTC**
Formalin specimens (n=20)	Minimum	13.0	1.4	2.6	1.0	0.9	66.0	4
	Maximum	35.0	2.5	5.8	1.9	1.7	89	7
	Mean	24.5	1.8	4.0	1.5	1.3	78.6	5.3
	SD	6.6	0.4	0.9	0.3	0.3	6.0	0.9

**Table 2.** Ranges, means and standard deviations (SD) in number of paragnaths in three *Nereis* species (r: right, l: left).

	Pharynx areas									
***Nereis oligohalina* (n=27)**	**I**	**II-r**	**II-l**	**III**	**IV-r**	**IV-l**	**V**	**VI-r**	**VI-l**	**VII–VIII**
Minimum	8	25	25	32	22	22	1	4	3	40
Maximum	15	38	37	71	45	39	2	4	4	47
Mean	11.7	31.0	30.4	49.1	31.5	31.4	1.04	4.0	3.9	43.9
SD	2.3	2.9	3.1	9.2	4.9	3.6	0.2	0.00	0.2	2.0
***Nereis garwoodi* (n=29)**	**I**	**II-r**	**II-l**	**III**	**IV-r**	**IV-l**	**V**	**VI-r**	**VI-l**	**VII–VIII**
Minimum	4	19	20	28	22	19	1	4	3	42
Maximum	19	42	40	59	43	38	1	4	4	46
Mean	10.7	30.3	30.5	44.6	31.0	30.1	1.0	4.0	3.9	44.0
SD	3.4	5.3	5.7	6.4	5.3	4.5	0.00	0.00	0.3	1.3
***Nereis confusa* sp. n. (n=30)**	**I**	**II-r**	**II-l**	**III**	**IV-r**	**IV-l**	**V**	**VI-r**	**VI-l**	**VII–VIII**
Minimum	4	30	28	39	34	35	1	3	3	42
Maximum	11	36	42	62	58	54	1	5	6	45
Mean	7.3	32.3	34.2	51.2	45.6	45.1	1	3.6	4.0	42.9
SD	2.1	2.3	3.9	8.1	8.9	6.6	0	0.7	0.9	1.1

In the case of *Nereis oligohalina*, fixation with 96% ethanol clearly affected the anterior portion of specimens with strong contraction of first segments, hence the tentacular cirri can reach more posterior chaetigers but without modifying their lengths, reaching up to chaetiger 14 (Table 1). Differences are not significant for L_10_, W_3_ and W_10_ (P = >0.05), but significant for L_3_ and rTC (P = <0.001, P = <0.0001). Maximum rTC for formalin specimens was preferred for the identification key because the data were less variable (Table 1). Nevertheless, these differences would not cause misidentification, because parapodial topology is not affected appreciably. Also pigmentation is very useful for recognizing the species.

**Figure 6.**
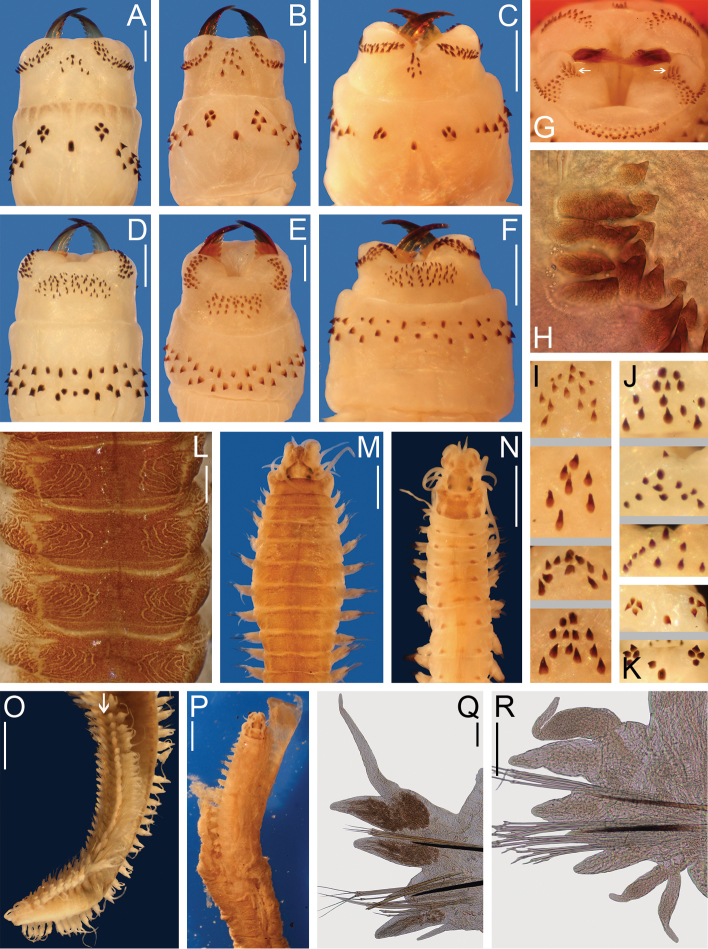
Variation of *Nereis* species studied. *Nereis oligohalina* **A, D, J–L, O, Q** from ECOSUR–OH–P0760. *Nereis garwoodi* **B, E** from paralectotype ECOSUR 0066; **I, M, R** from ECOSUR P2834. *Nereis confusa* sp. n. **C, F–H, N, P** from ECOSUR P2838. **A–C** Pharynges everted, dorsal view **D–F** Pharynges everted, ventral view **G** Pharynx everted, anterior view, showing merged paragnaths (arrows) **H** Close-up of merged paragnaths on area IV **I–J** Variations on area I **K** Variations on areas V and VI **L** Fingerprint-like pattern, dorsal view **M–N** Pigmentation patterns on anterior ends, dorsal view **O** Parapodial furrow in posterior end, lateral view (arrow indicates start) **P** Specimen on tube, dorsal view **Q** Parapodium from posterior chaetiger, anterior view **R** Ventral cirrus duplicated, chaetiger 5, anterior view. Scale bars: 0.5 mm (**A–F, L**); 1 mm (**M–P**); 0.1 mm (**Q–R**).

In the maxillary ring, area I showed the least variation (Fig. 6J), and in oral ring areas V and VI rarely vary in one paragnath only (Fig. 6K), such that these areas can be regarded as the most stable ones. The fingerprint-like pattern starts in chaetigers 10–11 (Fig. 6L); it is size-independent, but in smaller specimens this pattern is faint; however, as shown below it is absent in the two other species. The divergence between parapodial rami reported by Rioja (1946) is evident in posterior chaetigers but only in some specimens, forming a furrow (Fig. 6O). Glandular masses appear more visible and also on neuropodial ligules (Fig. 6Q); perhaps these glands fade in specimens fixed with formalin, such as the type material. In mature specimens, the natatory region starts in chaetiger 15–17 in males, and 24–25 in females.

##### Remarks.

*Nereis oligohalina* (Rioja, 1946) is considered as a widespread species and even amphiamerican, but this stems from taxonomic confusion and the lack of type material. Designation of a neotype for *Nereis oligohalina* was considered necessary because there are no type specimens and being a problematic species, there must be an objective definition for it (ICZN 1999, Art. 75.1). Consequently, a neotype has been selected, described and illustrated (ICZN 1999, Art. 75.3.3); this neotype fits the original description by Rioja (1946) (ICZN 1999 Art. 75.3.5). Because Rioja did not designate holotype, his material became syntypes and the species had two type localities (ICZN 1999 Art. 73.2.3, 76.1): Estero de Larios, Tecolutla, and El Cocal, Estero Casitas, both in Veracruz, Mexico. Although topotypic specimens from Estero Casitas are available, they are in poor condition, and therefore better specimens collected from nearby Actopan River were preferred once they were shown to conform to the same species (ICZN 1999, Recomm. 75A). The proposed neotype was collected in a similar environment and on oysters, as the original specimens (ICZN 1999, Art. 75.3.6); but the neotype locality is modified accordingly (ICZN 1999, Art. 76.3). The neotype was deposited in ECOSUR (ICZN 1999, Art. 75.3.7), including ‘paraneotypes’ and part of the additional material.

*Neanthes oligohalina* Rioja, 1946 was correctly transferred to *Nereis* by Hartman (1951) because there are notopodial homogomph falcigers in posterior chaetigers. Hartman (1951, 1954) suggested that *Nereis oligohalina* was ‘inseparable’ from *Nereis pelagica occidentalis* Hartman, 1945, but not synonymized. Salazar-Vallejo (1989) noted that although Rioja clearly recognized the presence of notopodial homogomph falcigers, he maintained the species under *Neanthes*, even in later publications (Rioja 1947, 1960, 1962).

The first synonymy involving these species was made by Pettibone (1956); she considered *Nereis pelagica occidentalis* different from *Nereis pelagica* and raised it to species level as Nereis (Nereis) occidentalis Hartman. Also, she regarded *Neanthes oligohalina* as a junior synonym of *Nereis occidentalis* being regarded as a variety. The detailed description provided by Pettibone allowed us to recognize differences in comparison to the Laguna Madre, Texas specimens. She recognized slight but important differences among these variants, mainly in paragnath number in areas V and VI; adding the relative size of neuropodial ligules in middle and posterior chaetigers. In the same work, she determined that *Nereis largoensis* Treadwell, 1931 was a junior synonym of *Nereis pelagica*, and that other material identified as *Nereis largoensis* based upon material examined by Treadwell corresponds to *Nereis occidentalis* (Pettibone 1956). We follow, however, González-Escalante and Salazar-Vallejo (2003), who concluded that these three species are not synonyms.

*Nereis oligohalina* differs from *Nereis occidentalis* in some diagnostic features. In *Nereis oligohalina* there are 8–15 paragnaths on area I and 1–2 on area V, whereas in *Nereis occidentalis* there are 2–3 paragnaths on area I and no paragnaths on area V. Further, in *Nereis oligohalina* neuropodial ventral ligules are 2–3 times longer than neuroacicular ligules, but in *Nereis occidentalis* neuropodial ventral ligules from posterior chaetigers are shorter than neuroacicular ones. Regarding chaetae, and as an additional difference, in *Nereis oligohalina* the notopodial homogomph falciger has the distal tooth less developed than in *Nereis occidentalis*.

Another synonymy was made by Day (1973), who regarded *Nereis pelagica occidentalis* Hartman and *Nereis occidentalis fide* McCloskey as junior synonyms of *Nereis falsa* de Quatrefages, 1865; however, in the list of synonyms of these two species he did not include Pettibone (1956), therefore *Nereis oligohalina* was not considered by him. *Nereis falsa*, has a rather complex or confusing delineation; according to Fauvel (1923) it differs from similar species by having different numbers of paragnaths in areas I and V, and different proportions in parapodial ligules. In fact, *Nereis falsa* is another species regarded as widely distributed and requires a critical revision and we could anticipate a restriction of its distribution to the Mediterranean region because its type locality is the Black Sea.

On the other hand, *Nereis oligohalina* has been recorded along American Atlantic coasts from northeastern Brazil, chiefly in ecological (community assemblages on *Spartina alterniflora* and mangroves), or population studies (secondary production and population dynamics), as well as part of taxonomic or genetic studies (Amaral et al. 2012). A detailed record was made by Santos and Lana (2003); unfortunately, a commentary and one plate was based upon specimens collected in Todos Los Santos Bay, and indicated that their material agrees with the original description regarding neuropodial ventral ligules in posterior parapodia, and the feature was less developed in specimens from other localities but were regarded as the same because the prostomial pigmentation and paragnath number remained constant (Santos and Lana 2003). Lana et al. (2006) however, considered their previous record as a probable misidentification requiring a revision. Liñero-Arana and Díaz (2007) recorded *Nereis oligohalina* from Venezuela in La Restinga Lagoon, Margarita Island, associated with *Crassostrea rhizophorae* (Guilding), and recognized that their specimens resembled Brazilian ones and differed from those described by Rioja, mainly in parapodial morphology. These two publications pointed out the need for a revisionary work, recognizing *Nereis oligohalina* as valid species, but that their specimens were probably not the same as those described from Mexico because of parapodial features; at least the specimens from South America were regarded as a different species that should be clarified elsewhere.

Records of *Nereis oligohalina* from the Eastern Tropical Pacific (Berkeley and Berkeley 1958, 1960) belong to a new species described below.

##### Habitat.

The species is associated with red mangrove *Rhizophora mangle* and with oysters. It has been found in Gulf of Mexico estuaries, including Tecolutla, Casitas-Nautla and Actopan (these estuaries have sand bars in their respective mouth rivers), and from coastal lagoons such as Mandinga and La Mancha. These systems have direct connection with the sea, some with seasonal closure of their mouths, with polyhaline to mesohaline waters (Lara-Domínguez et al. 2011).

The neotype and associated specimens were found in *Crassostrea virginica* (Gmelin) reef, in the Actopan river mouth. The specimens studied by Rioja (1946) from the Tecolutla estuary were found on mangrove roots covered by cirripedians, whereas specimens from the Casitas-Nautla estuary were collected between oysters (possibly *Crassostrea virginica*) and mytilids as *Ischadium recurvum* (Rafinesque) (reported as *Mytilus recurvatus* (sic) by Rioja); as Rioja indicated, *Nereis oligohalina* specimens cohabit with an *Alitta* species in the Actopan river mouth. This species has been reported as *Nereis occidentalis* Hartman, together with *Polydora websteri* Hartman, as epifauna of *Crassostrea virginica* (Ruiz-Guerrero and López-Portillo Guzmán 2006), and on *Rhizophora mangle* roots (Ruiz and López-Portillo 2014), from La Mancha.

##### Distribution.

Restricted to the southwestern Gulf of Mexico.

#### Nereis garwoodi

Taxon classificationAnimaliaPhyllodocidaNereididae

González-Escalante & Salazar-Vallejo, 2003

Figures 3 , 4 , 6B, E, M, I, R

Nereis garwoodi González-Escalante and Salazar-Vallejo 2003: 156–160, figs 1a–k, 2a–h.

##### Type material.

**Quintana Roo, Mexico.** Lectotype ECOSUR 0065 and paralectotypes ECOSUR 0066 (7), Chetumal (18°29'38.88"N, 88°17'22.89"W), Chetumal Bay, 1 m depth, in calcareous sedimentary rocks, mixed bottom, September 24 1999, Coll. L.E. González-Escalante, S.I. Salazar-Vallejo.

##### Additional material.

**Chetumal Bay, Quintana Roo, Mexico.** ECOSUR P2829 (3), Alacranes (18°34'28.51"N, 88°14'24.21"W), May 1 1999, Coll. LEGE, SISV. ECOSUR P2830 (14), Chetumal, May 3 1999, Coll. LEGE, SISV. ECOSUR P2831 (29), Chetumal, 21 May 1999, Coll. LEGE, SISV. ECOSUR P2832 (17), Alacranes (18°34'28.51"N, 88°14'24.21"W), May 21 1999, Coll. LEGE, SISV. ECOSUR P2833 (2), Luis Echeverría (18°39'04’’N 88°12'07’’W), May 21 1999, Coll. LEGE, SISV. ECOSUR P2834 (28), Chetumal (18°29'38.88"N, 88°17'22.89"W), June 30 1999, Coll. LEGE, SISV. ECOSUR P2835 (9), Chetumal (18°29'38.88"N, 88°17'22.89"W), August 27 1999, Coll. LEGE, SISV.

##### Type locality.

Chetumal Bay, Mexico, Caribbean Sea, on rocks in mixed bottoms, 1 m depth.

##### Description.

Lectotype complete (ECOSUR 0065), atokous female, damaged with incisions at level of chaetigers 1, 15, and 30. Body tapering, 36 mm long, 1.7 mm wide, 95 chaetigers. Body pale, pigmentation faint, brown rectangle present dorsally on middle of anterior chaetigers, striated, discoloring toward end of body, lateral pale lines in anterior chaetigers only, oocytes present. Prostomium with brown pigment along inner margins of palps, two lines extending from antennae toward anterior pair of eyes separated by a longitudinal pale area, and two oval lateral patches; peristomium slightly pigmented, pale lines present (Fig. 3A).

Prostomium 1.5 times longer than wide; antenna cirriform, slightly passing palps; eyes subequal, black, in a rectangle (Fig. 3A). Peristomium twice longer than first chaetiger; tentacular cirri with short ceratophores, left cirri broken; dorsal longer than ventral ones, posterodorsal ones reaching chaetiger 12 (Fig. 3A).

Pharynx everted, jaws pale brown with 13 rounded teeth, extending to base (Fig. 3I). Maxillary ring: I = 15 pyramids in diamond, II = 31–31 pyramids and cones in arc, III = 44 cones in an ellipse, IV = 35–35 pyramids in arc (Fig. 3A). Oral ring: V = 1 cone, VI = 4–4 pyramids in diamond, VII-VIII = 42 in two irregular rows, P-bars alternating with small pyramids in anterior-most row, pyramids and cones with similar size alternating in posterior-most row.

Parapodial cirri pattern: Dorsal cirri longer than upper dorsal ligules throughout body; basally inserted on anterior region, displaced medially in midbody region, becomes subdistal in posterior chaetigers. Ventral cirri longer than neuropodial ligules in a few anterior chaetigers, progressively reduced throughout body; basally inserted on anterior region, barely migrating ventrally throughout body.

First two chaetigers uniramous, remaining ones biramous. In uniramous parapodia (Fig. 3D), dorsal cirri basal, slightly longer than notopodial ligules. Dorsal and neuropodial ventral ligules subequal, digitate, three times longer than neuroacicular ligules; neuroacicular ligules subconical, postchaetal lobes rounded. Ventral cirri subequal to neuropodial ventral ligules; both dorsal and ventral cirri with similar length and width.

In anterior parapodia (Fig. 3E), dorsal cirri medial, longer than notopodial ligules, extending beyond them. Notopodial dorsal ligules subconical, slightly longer than ventral ones; notopodial ventral ligules subconical, slightly longer than neuroacicular ligules, notoacicular papillae very conspicuous. Neuroacicular ligules subconical, subequal to ventral ones, postchaetal lobes rounded, slightly shorter than neuroacicular ligules; neuropodial ventral ligules digitate, basally attached to neuroacicular ligules. Ventral cirri shorter than neuropodial ventral ligules; dorsal cirri twice wider than ventral ones.

In middle parapodia (Fig. 3F), dorsal cirri medial, as long as notopodial ligules, extending beyond them. Notopodial dorsal and ventral ligules subequal, subconical, notoacicular papillae inconspicuous. Neuroacicular ligules subconical, slightly shorter than remaining ones, postchaetal lobes rounded, shorter than neuroacicular ligules; neuropodial ventral ligules digitate, basally attached to neuroacicular ones. Ventral cirri half as long as neuropodial ligule; both dorsal and ventral cirri with similar width.

In posterior parapodia (Figs 3G, H), dorsal cirri medial, slightly longer than notopodial ligule. Notopodial dorsal ligules become broad, longer than ventral ones; notopodial ventral ligules become large, 2–3 times longer than neuroacicular ligules, notoacicular papilla inconspicuous. Neuroacicular ligules subconical, half as long as neuropodial ventral ones, postchaetal lobes inconspicuous; neuropodial ventral ligules digitate, basally attached to neuroacicular ligules. Ventral cirri up to half as long as neuropodial ligule; dorsal and ventral cirri with similar width. Glandular masses conspicuous on notopodial ligules.

In anterior and midbody parapodia, notochaetae homogomph spinigers; neurochaetae homogomph spinigers and heterogomph falcigers in supra-acicular fascicles, heterogomph spinigers and falcigers in sub-acicular fascicles. In posterior parapodia, notochaetae homogomph spinigers and falcigers; neurochaetae as in anterior parapodia. Number of chaetae decreasing toward posterior end.

Notopodial homogomph spinigers pectinate, teeth decreasing in size distally (Fig. 3O). Notopodial homogomph falcigers with sigmoid blade, pectinate, distal tooth incurved, fused to blade (Fig. 3C). Neuropodial homogomph spinigers pectinate or basally serrate (Fig. 3J), heterogomph spinigers pectinate (Fig. 3P); both with teeth decreasing in size distally. Neuropodial heterogomph falcigers pectinate, distal tooth incurved, fused to blade, supra-acicular slightly broader than sub-acicular (Fig. 3L–N); supra-acicular falcigers narrow in midbody chaetigers, becoming broad posteriorly (Fig. 3L, M).

Pygidium without modification; anal cirri cirriform, as long as last 4–5 segments (Fig. 3B).

##### Epitokes.

Paralectotype fully transformed male (ECOSUR 0066) complete, body tapering, 9 mm long, 1 mm wide, 46 chaetigers; paralectotype partially transformed female (ECOSUR 0066) incomplete, body tapering, 16 mm long, 2 mm wide, 63 chaetigers; fully transformed female (ECOSUR P0066) complete, body tapering, 12 mm long, 1.6 mm wide, 63 chaetigers. All with body yellowish with brown pigmentation present dorsally on first quarter of body, discoloring toward midbody chaetigers (Fig. 4E, F). Prostomium and peristomium with pigmentation similar to atokes (Fig. 4A, B).

Prostomium longer than wide; antennae cirriform, as long as palps; eyes black, subequal, in a rectangle, three times larger than antennal basal width (Fig. 4A, B). Peristomium twice length of first chaetiger; tentacular cirri with short ceratophores, dorsal tentacular cirri longer than ventral ones, posterodorsal reaching to chaetiger 9 in male, 13 in female (Fig. 4A, B).

Male with pharynx everted, jaws amber with 9 teeth. Maxillary ring: I = 6 pointed cones in triangle, II = 19–20 pointed cones in arc, III = 28 pointed cones in rectangle, IV = 22–19 pointed cones in arc. Oral ring: V = 1 pointed cone, VI = 4–3 pyramids in diamond, VII-VIII = 42 in two irregular rows, P-bars alternating with small pyramids in most-anterior row, pyramids alternating with cones with similar size in most-posterior.

Male body divided into two regions (Fig. 4E); pre-natatory region includes chaetigers 1–16, natatory region from chaetiger 17 to end of body. Fully transformed female body with two regions; pre-natatory includes chaetigers 1–26, natatory region from chaetiger 27 to end of body (Fig. 4F). Partially transformed female divided in two inconspicuous regions, lamellae start in chaetiger 25.

Parapodial cirri pattern: Anterior parapodia with dorsal cirri modified in chaetigers 1–7 in males, 1–5 in females; ventral cirri modified in chaetigers 1–5 in males, 1–4 in females. Dorsal cirri subequal to upper dorsal ligules in anterior chaetigers, become subequal throughout body; basally inserted in most-anterior region, displaced medially throughout body. Ventral cirri shorter than neuropodial ligules in unmodified chaetigers, subequal in modified region; basally inserted in anterior region, barely migrating ventrally throughout body.

Chaetigers 1–2 with uniramous (Fig. 4G, L), modified dorsal cirri basal, subpyriform in males, cattail-like in females, subequal to dorsal ligules. Dorsal and neuropodial ventral ligules subequal, subconical, twice longer than wide in male, 1.5 times longer than wide in female. Neuropodial lobe subconical, shorter than dorsal ligules; postchaetal lobes rounded. Modified ventral cirri shorter than neuropodial ventral ligules; dorsal and ventral cirri subequal with similar width and length.

Chaetigers 3–7 in males (Fig. 4H) and 3–5 in females (Fig. 4M) with slightly modified biramous parapodia. Modified dorsal cirri medial, cattail-like, slightly longer than notopodial ligules, extending beyond them; broader section as long as narrower one. Notopodial dorsal ligules subconical, slightly longer than notopodial ventral ligules; notopodial ventral ligules subconical, twice longer than neuroacicular ligules, notoacicular papillae conspicuous. Neuroacicular ligules subconical, postchaetal lobes rounded; neuropodial ventral ligules digitate, longer than neuroacicular ones. Modified ventral cirri cattail-like, subulate in chaetigers 6–7 in males and 5 in females, shorter than neuropodial ventral ligules.

Parapodial proportions as in atokous from chaetigers 8–16 in male and 6–26 in female (Fig. 4I, N).

Remaining parapodia modified (Fig. 4J, K, O, P). Dorsal cirri medial, subulate, ventral margins sinuate in males only, subequal to notopodial ventral ligules; basal lamellae large in males, small in females, increasing size toward posterior chaetigers and decreasing in most-posterior ones.

Notopodial dorsal ligules subconical, longer than ventral ones in male, subequal in female; notopodial ventral ligules subconical, developing a large ventral lamella in males only, with a round projection. Neuroacicular ligules subconical, shorter than notopodial ventral ones; postchaetal lobes developing into flabellate lamellae with a round projection in dorsal edge in males, small lamellae in females, progressively increasing in size and decreasing in far posterior segments; neuropodial ventral ligules digitate, basally attached to neuroacicular ones. Ventral cirri subulate, slightly longer than neuroacicular ligules, with two basal lamellae of different sizes; dorsal cirri wider than ventral ones.

Prenatatory region with noto- and neurochaetae as in atokes, homogomph falcigers not observed. In natatory region, notochaetae and neurochaetae sesquigomph chaetae with finely serrated, paddle-like blades; atokous chaetae not completely replaced in specimens of either sex, homogomph falcigers observed in male (Fig. 4J, K, O, P).

Pygidium modified, anus surrounded by rosette of papillae in male, unmodified in female (Fig. 4C, D); anal cirri cirriform, as long as last 5–6 segments (Fig. 4C, D).

##### Variation.

The results of the analysis of body variation and analysis of paragnath numbers are summarized in Tables 1 and 2. The width measurements reported here differ from the original description because chaetiger width without parapodia was used, instead of measuring them including parapodia. The arrangement and number of paragnaths have similar ranges as those reported for *Nereis oligohalina* (Fig. 6B, E; Table 2); however, area I has a larger range, and the arrangement is somewhat variable, often in a triangle (Fig. 6I). Also, paragnaths are more robust than in *Nereis oligohalina* and *Nereis confusa* sp. n.

Regarding pigmentation, the striated rectangle seen in lectotype is more conspicuous in some specimens (Fig. 6M), which is also sometimes present in *Nereis oligohalina*, but the color is much more intense whereas the fingerprint-like pattern of the latter species was not observed. In mature specimens, the natatory region starts from chaetiger 17 only in males and 25–27 in females, which differs from the original description (22 in males and 21 in females). One specimen presented a duplicated ventral cirrus, but it was regarded as abnormal (Fig. 6R).

##### Remarks.

González-Escalante and Salazar-Vallejo (2003) indicated that they had six atokes and two epitokes as syntypes. Five atokous syntypes were expected to be sent to four foreign museums, but were never dispatched. Further, these syntypes were not formally deposited and labeled, and parts of the descriptions and illustrations are too imprecise to enable separation of *Nereis garwoodi* from *Nereis oligohalina*.

In an attempt to redefine the species, a lectotype has been selected (ICZN 1999, Art. 74.1) to avoid future confusion; although the syntype series has better preserved specimens, the lectotype matches the original description and illustration, and was therefore preferred (ICZN 1999, Recomm. 74B). In order to ensure their validity, the term has been introduced in the material section and in the description (ICZN 1999, Art. 74.7.1, 74.7.3), and the lectotype has been described, illustrated and their data updated for its recognition (ICZN 1999, Art. 74.7.2, Recomm. 74C, 74E); the remaining syntypes are regarded as paralectotypes (ICZN 1999, Recomm. 74F). These specimens are deposited in ECOSUR.

*Nereis garwoodi* is closely allied with *Nereis oligohalina*, but they differ in some features in both atokous and epitokous forms, and in their habitats. In atokes, *Nereis garwoodi* never shows the dark brown coloration nor the fingerprint-like pattern found in *Nereis oligohalina*. The ranges of paragnath numbers of both species overlap and therefore are not useful to separate them, and the relative length of tentacular cirri would be useful if fixation method is the same (Table 1).

In *Nereis garwoodi*, both dorsal and neuropodial ventral ligules are twice as long as neuroacicular ligules in uniramous chaetigers, whereas in *Nereis oligohalina* these are subequal and slightly longer, respectively. Also, in *Nereis garwoodi* the neuropodial postchaetal lobes are visible in the anterior and midbody only, whereas in *Nereis oligohalina* they are visible throughout body.

Further, *Nereis garwoodi* has notopodial ventral ligules twice as long as neuropodial ventral ones in posterior chaetigers, whereas in *Nereis oligohalina* these ligules are subequal to each other; further, in *Nereis oligohalina* neuropodial ventral ligules are medially attached in posterior chaetigers, whereas in *Nereis garwoodi* they are basally attached throughout body. Moreover, in *Nereis garwoodi* notopodial homogomph falcigers have more teeth and they are narrower than in *Nereis oligohalina*; also, in *Nereis garwoodi* the blades of supra-acicular heterogomph falcigers become broader and shorter in posterior chaetigers, but this modification is not present in *Nereis oligohalina*.

In epitokes, *Nereis garwoodi* has modified, cattail-like dorsal cirri present in biramous chaetigers with the basal sections as long as distal ones, whereas in *Nereis oligohalina* basal sections are longer. Also, in general *Nereis garwoodi* have better developed lamellae in natatory chaetigers than *Nereis oligohalina*, especially the basal lamellae of the dorsal cirri, the lamellae of both notopodial ventral and neuroacicular ligules. Moreover, epitokal transformation is more pronounced in females of *Nereis garwoodi* than in females of *Nereis oligohalina*. On the other hand, *Nereis garwoodi* is associated with calcareous rocks, while *Nereis oligohalina* is associated with reef-building bivalves and the mangrove *Rhizophora mangle*.

##### Habitat.

Chetumal Bay is a semi-closed, dynamic system linked to the Caribbean Sea by several freshwater tributaries, having a salinity gradient ranging 7–18 practical salinity units (psu) (Carrillo et al. 2009). The species bores into calcareous sedimentary rocks, building mucous tubes, and has been regarded as a sedentary herbivore (González-Escalante and Salazar-Vallejo 2003); to obtain the specimens, rocks must be broken. Although the Bay has extensive zones of mangroves, *Nereis garwoodi* has never been found among them.

##### Distribution.

Apparently restricted to Chetumal Bay. González-Escalante and Salazar-Vallejo (2003) report a gradient of decreasing abundance from the southern to the northern regions of the bay, probably related to organic matter load.

#### Nereis confusa sp. n.

Taxon classificationAnimaliaPhyllodocidaNereididae

http://zoobank.org/5048FF4A-0F6A-4B03-BCF5-352F41EDBC39

Figures 5 , 6C, F–H, N, P

Nereis (Neanthes) oligohalina Berkeley and Berkeley 1958: 402 (*non* Rioja, 1946).
Nereis oligohalina Berkeley and Berkeley 1960: 359 (*non* Rioja, 1946).

##### Type material.

**Gulf of California**, **Baja California Sur.** Holotype ECOSUR 0174 and paratypes ECOSUR 0175 (5), Bahía de La Paz (24°08'38.68"N, 110°20'44.40"W), March 1 2004, 70 m from shore, on wrinkled pen shell *Pinna rugosa*, sponges, PVC tube, and filamentous green algae, Coll. M.A. Tovar-Hernández, P. Salazar-Silva.

##### Additional material.

**Gulf of California, Baja California**. ECOSUR P2836 (16), Bahía de Los Ángeles (28°58'6.72"N, 113°32'43.24"W), Gulf of California, March 17 1985, on *Atrina maura*, Coll. E. Aguirre, C. Garza. **Gulf of California, Sinaloa.** ECOSUR P2837 (10), Estero el Yugo (23°18'8.30"N, 106°29'0.53"W), Mazatlán, February 24 2004, 50 cm depth, fine sediment, on filamentous green algae on mangrove roots, Coll. S. Rendón-Rodríguez, Nuri M., M.A. Tovar-Hernández, P. Salazar-Silva. **Baja California Sur.** ECOSUR P2838 (32), same data as holotype. ECOSUR P2839 (2), Bahía La Paz (24°12'6.51"N, 110°17'59.26"W), Gulf of California, March 2 2004, 1 m depth, on basalt rocks, sponges and algae, Coll. M.A. Tovar-Hernández, P. Salazar-Silva.

##### Etymology.

The specific name (L. *confusa*: confused, perplexed, troubled) indicates an earlier problematic delineation of the species; it is a noun in apposition.

##### Description.

Holotype complete (ECOSUR 0174), atokous female. Body tapering, 34 mm long, 1.7 mm wide, 81 chaetigers, inmature. Body yellowish, reddish brown pigmentation present dorsally on first quarter of body as three spots pattern, two lateral ones, and the other less pigmented, middorsal, forming discontinuous transverse bands up to chaetiger 10, remaining segments pale; lateral pale lines in anterior chaetigers only (Fig. 5A). Prostomium with pigmentation brown on inner margins of palps and with two oval patches (Fig. 5A); peristomium dorsally pigmented, variegated (Fig. 5A), with very short pale lines on posterior margin.

Prostomium longer than wide; antennae cirriform, extending beyond palps; eyes subequal, black, in a rectangle (Fig. 5A). Peristomium twice longer than first chaetiger; tentacular cirri with short ceratophores, dorsal cirri longer than ventral ones, posterodorsal ones reaching to chaetiger 5 (Fig. 5A).

Pharynx dissected, jaws with 8 teeth, restricted to anteromedial edge, light brown (Fig. 5I). Maxillary ring: I = 5 cones in rectangle, II = 30–30 cones in arc, III = 49 cones in an ellipse, IV = 41–36 cones and some merged, in sigmoidal. Oral ring: V = 1 cone, VI = 5–5 cones in round, VII-VIII: 42 in two irregular rows, P-bars and small cones alternating in most anterior row, pyramids and small cones in most-posterior row.

Parapodial cirri pattern: Dorsal cirri longer than upper dorsal ligules throughout body; basally inserted on anterior region, displaced medially on midbody and posterior regions. Ventral cirri as long as neuropodial ligules in a few anterior chaetigers, progressively reduced throughout body; basally inserted in anterior region, migrating ventrally throughout body.

First two chaetigers uniramous, remaining biramous. In uniramous parapodia (Fig. 5D), dorsal cirri basal, slightly longer than dorsal ligules. Dorsal ligules subconical; neuroacicular ligules subconical, subequal to dorsal ligules; neuropodial ventral ligules digitate, shorter than neuroacicular ligules. Ventral cirri subequal than neuropodial ligule; dorsal cirri slightly wider than ventral ones.

In anterior parapodia (Fig. 5E), dorsal cirri medial, as long as notopodial dorsal ligules, extending beyond them. Notopodial dorsal ligules subconical; notopodial ventral ligules globose, subequal to dorsal ones, notoacicular papillae conspicuous. Neuroacicular ligules subconical, postchaetal lobe rounded, subequal than neuroacicular ligules; neuropodial ventral ligules digitate, shorter than neuroacicular ones. Ventral cirri shorter than neuropodial ligule; dorsal cirri slightly wider than ventral ones.

In midbody and posterior parapodia (Figs 5F–H), dorsal cirri medial, subequal to notopodial dorsal ligules. Notopodial dorsal and ventral ligules subequal, slightly enlarged in posterior parapodia, subconical, longer than wide, notoacicular papillae conspicuous in middle parapodia. Neuroacicular ligules subconical, postchaetal lobes rounded, shorter than notopodial ligules; neuropodial ventral ligules digitate, slightly shorter than neuroacicular ones. Ventral cirri shorter than neuropodial ventral ligules; dorsal cirri slightly wider than ventral ones (Fig. 5F–H).

In anterior and midbody parapodia, notochaetae homogomph spinigers; neurochaetae homogomph spinigers and heterogomph falcigers in supra-acicular fascicles, heterogomph spinigers and falcigers in sub-acicular fascicles. In posterior parapodia, notochaetae homogomph spinigers and falcigers; neurochaetae as in anterior parapodia.

Notopodial homogomph spinigers pectinate, teeth decreasing in size distally. Notopodial homogomph falcigers pectinate, 9 teeth, distal tooth stout, incurved, fused to blade (Fig. 5C). Neuropodial homogomph spinigers basally serrate (Fig. 5J), heterogomph spinigers pectinate or serrate (Fig. 5K); both with teeth decreasing in size distally. Neuropodial heterogomph falcigers pectinate, distal tooth incurved, fused to blade, very conspicuous (Fig. 5L–N); in posterior parapodia with short (Fig. 5M) or long (Fig. 5N) blades in both fascicles, missing in most chaetae.

Pygidium with broad margin, anus crenulated; anal cirri cirriform, as long as last 3–4 segments (Fig. 5B).

##### Variation.

The results of the analysis of body variation and paragnath numbers are summarized in Table 1 and 2. The arrangement and number of paragnaths is similar to that in *Nereis oligohalina* and *Nereis garwoodi*, but in *Nereis confusa* sp. n. paragnaths in areas III and IV are more numerous than in the other two species (Fig. 6C, F; Table 2), and cones have rounded tips; further, *Nereis confusa* sp. n. is the only species of the three with merged paragnaths.

All specimens examined show the same dorsal spotted pigmentation, but in some, especially the largest specimen, the middorsal spot disappears and only two discontinuous lines are visible along chaetigers 1–10; fingerprint-like or striated patterns were not observed. In mature specimens, the transformation starts in chaetiger 18 in males, 22 in females as previously noticed by Berkeley and Berkeley (1958, 1960).

##### Remarks.

*Nereis confusa* sp. n. has been recorded as *Nereis oligohalina*; however, there are several differences between these two species. In *Nereis confusa* sp. n. the spotted pigmentation pattern extends up to chaetigers 10–14, and the jaws have 8 teeth restricted to the distal part of its inner edge, whereas in *Nereis oligohalina* the pale areas are replaced by fingerprint-like patterns from chaetiger 11, and its jaws have 11 teeth along its inner edge. Further, in *Nereis confusa* sp. n., both notopodial ligules and neuroacicular ligules are subequal to, or slightly longer than, neuropodial ventral ligules in midbody and posterior parapodia, whereas in *Nereis oligohalina*, they are twice as long as the neuroacicular ligules in midbody and posterior parapodia. On the other hand, *Nereis confusa* sp. n. has falcigers with broad blades, whereas in *Nereis oligohalina* they are narrower. Furthermore, in *Nereis confusa* sp. n. distal tooth of notopodial homogomph falciger is short and well developed, whereas in *Nereis oligohalina* it is longer and weakly developed.

The first records for *Nereis confusa* sp. n. (as *Nereis oligohalina*) from the Mexican Pacific were made by Berkeley and Berkeley; first, they reported Nereis (Neanthes) oligohalina males from Hipolito Bay (Berkeley and Berkeley 1958), and males and females from La Paz (Berkeley and Berkeley 1960). They argued that prostomium, anterior chaetigers and arrangement of paragnaths all matched Rioja’s descriptions. In their brief comments, they indicated the start of the modified region or first epitokous parapodium (17 in males, 22 in females), and a spotted pattern of pigmentation in males. These features match with *Nereis confusa* sp. n. rather than *Nereis oligohalina*.

Rioja (1962) cited Berkeley and Berkeley (1958), and recorded *Nereis confusa* sp. n. (as *Nereis oligohalina*) from El Mogote, Ensenada de La Paz, Baja California Sur; he mentioned a slight discrepancy in number of paragnaths in area I, and that paragnaths in the periphery of area III were larger than the rest of the group forming a borderline; also, he regarded the glandular parapodial masses as typical. Despite the fact that he did not provide more information, we regard his specimens as belonging to *Nereis confusa* sp. n. Other Mexican Pacific reports of *Nereis pelagica occidentalis* by Bastida-Zavala (1993, 1995) from nearby localities might also be conspecific.

Dean (2001) reported *Nereis oligohalina* from Pacific Costa Rican coasts, noticed the problems in the taxonomic history of the species, and regarded it as different from *Nereis occidentalis*. According to his description Costa Rican specimens differ from *Nereis oligohalina* in the number of paragnaths, mainly in areas I, III and IV. Also, in his specimens the longest tentacular cirri reached chaetiger 3, and the notopodial dorsal and neuropodial ventral ligules were subequal to or shorter than notopodial ventral and neuroacicular ligules throughout body, whereas in *Nereis oligohalina* the longest tentacular cirri reaches chaetiger 7, and their ligules are larger in midbody and posterior chaetigers. Likewise, Costa Rican specimens resemble *Nereis confusa* sp. n. and probably belong to the same species and this might also include the record from Cocos Island (Dean et al. 2012). Nevertheless, these records cannot be assigned to *Nereis oligohalina* unequivocally until specimens are evaluated.

##### Habitat.

Holotype found on wrinkled penshell *Pinna rugosa* Sowerby, 1835, sponges and filamentous green algae; other specimens were found in sponges and green algae near the type locality. Specimens from Bahía de Los Ángeles were associated with another penshell, *Atrina maura* (Sowerby, 1835), and specimens from Estero El Yugo were found on filamentous green algae on mangrove roots. Bastida-Zavala (1995) found specimens on corals.

##### Distribution.

Gulf of California, Eastern Pacific coasts of Mexico. Probably extends to Costa Rica, in shallow water.

##### Reproduction patterns and dispersal in *Nereis* species studied.

Some authors have emphasized the utility of reproductive patterns in taxonomy for species discrimination in closely related taxa (Smith 1958, Clark 1977). Also, strategies for larvae survival would be relevant, especially because they determine larval transport and its dispersal potential. An interesting strategy is when only males form epotikes and females remain atokous or are barely modified, as in *Alitta virens* (Sars, 1835) and *Websterinereis glauca* (Claparède, 1870). In *Alitta virens*, males form epitokes but females remain atokous or present very slight changes, also females spawn into or in the opening of their burrows (Bass and Brafield 1972). In *Websterinereis glauca*, female transformation is reduced, and females produce mucous tubes to deposit and incubate their eggs, while males can swarm (Pettibone 1971).

Early studies considered that *Nereis garwoodi* presented a similar reproductive mode as *Alitta virens* or *Websterinereis glauca*, because the paralectotype female of *Nereis garwoodi* has a slight transformation; however, after further revision of additional material, a fully transformed female was found. We have no further details about its capacity for building mucous tubes or if females do not emerge to the water column or if gametes are retained in tubes.

In the material available of *Nereis oligohalina*, no completely transformed females were encountered, but perhaps with further sampling efforts they may appear. Another important consideration is the reduced size of males compared to females, even in the same sample; this has been noted for *Hermodice diversicolor* (Bartels-Hardege and Zeeck 1990). In this case, *Nereis oligohalina* males can swarm while females remain inside cavities, as in *Alitta virens* and *Websterinereis glauca*; the fact that there are many large females filled with oocytes points toward this direction.

Reproduction modes in estuarine species play a crucial role in their dispersal because the formation of planktonic larvae can determine their distribution range. Bilton et al. (2002) proposed two life-cycle models for estuarine species that have larvae: export vs retention strategies. In the former, the adults respond to physical or biological factors by releasing gametes or larvae in the lower estuary; larvae are driven out from the estuary, mainly by tides, and later juveniles or adults return to the estuary. In the retention strategy, adults release their gametes or larvae in the upper estuarine areas, then they undergo early development in middle estuary; there, larvae have vertical migrations during circadian ebb-flood tidal regimes such that larvae are not exported but retained within the estuary (Bilton et al. 2002).

Based on the above, we hypothesize that *Nereis oligohalina* has an export strategy; it could disperse thanks to surface currents running parallel to the continental margin, and this would explain its presence along Gulf of Mexico estuaries. Similarly, distribution of *Nereis confusa* sp. n., with mainly marine habitats, could be due to current patterns along the Gulf of California.

However, sometimes the distribution patterns cannot be explained by currents and tidal dynamics. For example, De Jesús-Flores et al. (2015) determined that *Laeonereis nota* (Treadwell, 1941), described for Galveston, Texas, is also present in Chetumal Bay; *Laeonereis nota* spawn into their burrows, limiting their dispersal by currents. The explanation for this discontinuous distribution lies in passive dispersal through migratory birds, because they use nereidids as food (De Jesús-Flores et al. 2015). Similarly *Nereis garwoodi* could have a classical retention strategy, but a wide, fragmented distribution caused by migratory birds.

##### Further considerations.

The present study demonstrates the need to encourage redescriptions of closely related and widely distributed species and, should it be necessary, the establishment of new species if there are conspicuous morphological differences.

Further, clarifying species delineation and distribution are urgent because they are essential for biogeography and phylogenetics. Reuscher and Shirley (2014) studied the distribution patterns of polychaetes from the Gulf of Mexico; a recent species list was used (Fauchald and Solís-Weiss 2009) and current taxonomy verified in WoRMS (Read and Fauchald 2015). They found that among all species recorded, 32% were cosmopolitan, 15% Pan-American and 9% Pan-Atlantic (*Nereis oligohalina* was regarded as Pan-American, which is incorrect as shown above). They concluded: “Most polychaete families are in need of global and regional revisions. Clear species boundaries have to be established by means of taxonomic research based on morphology and genetic analyses. Geographical ranges of species should be revised in order to eliminate false conclusions about distributions of species.”

### Key to species of *Nereis* from the Grand Caribbean Region

(Modified from González-Escalante and Salazar-Vallejo 2003)

**Table:** 

1	Area V without paragnaths	**2**
–	Area V with paragnaths	**9**
2(1)	Parapodial ligules long, slender; ceratophores distinct	***Nereis caymanensis* Fauchald, 1977**
–	Parapodial ligules not enlarged; ceratophores indistinct	**3**
3(2)	Notopodia with prechaetal lobes	***Nereis goajirana* Augener, 1933^1^**
–	Notopodia without prechaetal lobes	**4**
4(3)	Dorsal cirri shorter than notopodial ligules; notopodial homogomph falcigers with oval blades	***Nereis grayi* Pettibone, 1956**
–	Dorsal cirri subequal or longer than notopodial ligules; notopodial homogomph falcigers with falcate blades	**5**
5(4)	Paragnaths on area I absent; notopodial homogomph falcigers with bifid blade	***Nereis panamensis* Fauchald, 1977**
–	Paragnaths on area I present, notopodial homogomph falcigers with entire blade	**6**
6(5)	Area VII-VIII with few paragnaths, usually 5–7	**7**
–	Area VII-VIII with numerous paragnaths, more than 40	**8**
7(6)	Longest tentacular cirri reaching chaetiger 7; area VI usually with 9 paragnaths	***Nereis riisei* (Grube & Ørsted *in* Grube, 1858)^2^**
–	Longest tentacular cirri reaching chaetiger 4; area VI usually with 3 paragnaths	***Nereis allenae* Pettibone, 1956^3^**
8(6)	Notopodial homogomph falciger with numerous teeth, distal tooth recurved, one quarter of blade embedded in shaft	***Nereis occidentalis* Hartman, 1945**
–	Notopodial homogomph falciger with few teeth, without distal tooth, one-half of blade embedded in shaft	***Nereis pelagica* Linnaeus, 1758^4^**
9(1)	Notopodial homogomph falcigers with cutting edge smooth	***Nereis largoensis* Treadwell, 1931^5^**
–	Notopodial homogomph falcigers with cutting edge denticulate	**10**
10(9)	Dorsal pigmentation as a striated pattern, usually along first 10 chaetigers; tentacular cirri reaching beyond chaetiger 7	**11**
–	Dorsal pigmentation different; tentacular cirri reaching up to chaetiger 7	**12**
11(10)	Area I with paragnaths in an oval; longest tentacular cirri reaching up to chaetiger 9	***Nereis rigida* Grube & Ørsted *in* Grube, 1858^6^**
–	Area I with paragnaths in a triangle; longest tentacular cirri reaching up to chaetiger 12	***Nereis garwoodi* González-Escalante & Salazar-Vallejo, 2003**
12(10)	Dorsal pigmentation with fingerprint-like pattern from chaetigers 10–11; notopodial ligules enlarged in posterior chaetigers	***Nereis oligohalina* (Rioja, 1946)**
–	Dorsal pigmentation with spotted pattern; notopodial ligules with similar proportions along body	***Nereis confusa* sp. n.^7^**

**Additional comments.** Several species recorded from the Grand Caribbean are questionable, such as *Nereis falcaria* (Willey, 1905), *Nereis jacksoni* Kinberg, 1866, *Nereis victoriana* Augener, 1918, *Nereis falsa* de Quatrefages, 1865, *Nereis callaona* (Grube, 1857) and *Nereis lamellosa* Ehlers, 1868; consequently, they were not included in the key because their type localities are distant and different form the tropical American conditions. Only *Nereis pelagica* was included in order to contrast it with *Nereis occidentalis*, but its records from the Grand Caribbean Sea might belong to a different species.

## Supplementary Material

XML Treatment for Nereis

XML Treatment for Nereis oligohalina

XML Treatment for Nereis garwoodi

XML Treatment for Nereis confusa

## Footnotes

1 This species has not been found recently (Báez and Ardila 2003).

2 It has a doubtful amphiamerican status and there is no recent redescription. Here, descriptions by Renaud (1956) with specimens from Miami, and by Hoagland (1919) for *Nereis glandulata* from Porto Rico, were used. Hartman (1956) considered *Nereis glandulata* as a junior synonym of *Nereis arroyensis* Treadwell, 1901, but they differ in number of paragnaths on area VII-VIII.

3 De León-González et al. (1999) detailed the arrangement of paragnath number of *Nereis allenae* Pettibone, 1956.

4 After Chambers and Garwood (1992).

5 In the original description of *Nereis largoensis* as *Nereis brevicirrata*, Treadwell (1929) reported area V without paragnaths and homogomph falciger with lenticular blades. However, Hartman (1956) found one paragnath on area V and homogomph falciger six times longer than wide.

6 Augener (1906) introduced the species name for the Caribbean fauna; however, Grube (1858) used a single specimen from Pacific Costa Rica (Puntarenas). Here the original description by Grube was used, and it was included as a contrast for *Nereis confusa* sp. n.

7 Distributed in the Mexican Pacific, often confused with *Nereis oligohalina*.
